# 填充吸附剂微萃取技术及其在微小体积样品萃取应用中的研究进展

**DOI:** 10.3724/SP.J.1123.2020.04024

**Published:** 2021-03-08

**Authors:** Jianan WEI, Molin QIN, Junchao YANG, Liu YANG

**Affiliations:** 国民核生化灾害防护国家重点实验室, 北京 102205; State key Laboratory of Nuclear, Biological and Chemical Protection for Civilian, Beijing 102205, China; 国民核生化灾害防护国家重点实验室, 北京 102205; State key Laboratory of Nuclear, Biological and Chemical Protection for Civilian, Beijing 102205, China; 国民核生化灾害防护国家重点实验室, 北京 102205; State key Laboratory of Nuclear, Biological and Chemical Protection for Civilian, Beijing 102205, China; 国民核生化灾害防护国家重点实验室, 北京 102205; State key Laboratory of Nuclear, Biological and Chemical Protection for Civilian, Beijing 102205, China

**Keywords:** 填充吸附剂微萃取, 样品基质, 微小体积样品, 样品前处理, 综述, microextraction by packed sorbent (MEPS), sample matrix, microvolume sample, sample pretreatment, review

## Abstract

微萃取技术是分析化学领域发展迅速,且已经得到广泛应用的样品前处理技术。填充吸附剂微萃取(MEPS)是一种微量固相萃取技术,使用微量的吸附剂填充于微量注射器,通过反复抽推方式使样品多次流经吸附剂以完成样品吸附萃取过程,萃取后的样品可直接用于色谱分析。典型的MEPS萃取设备包括MEPS注射器和MEPS吸附床(BIN)。MEPS优化的主要因素为MEPS处理过程的参数,包括样品流速、样品量与样品萃取循环次数,吸附剂及淋洗、洗脱溶剂的种类和体积,还需要考虑样品基质对MEPS性能的影响和样品残留和重复使用问题。MEPS中最重要的部分是吸附剂,主要有商品化的MEPS吸附剂,包括硅基的Silica、C18、C8等,碳材料的Hypercarb和聚苯乙烯聚合物类的SDVB、HDVB吸附剂等。研究用的吸附剂包括分子印迹材料、限进分子印迹材料、碳基材料、导电聚合物类材料、改性硅基材料及共价-有机骨架材料等。MEPS结合多种分析仪器已经成功应用于从不同基质中提取单一或多种分析物,所涵盖基质包括生物样品(尿液、唾液、血浆或血液)、河流水体或生活污水以及几种食品和饮料。MEPS处理复杂生物基质样品时,通常需要稀释样品、除蛋白质等预处理。MEPS具有需要样品体积小、操作快速等特点,在生物基质样品分析中有望得到更广泛的使用。在环境样品中,该技术可与现场便携仪器联用,未来将有望在现场进行快速检测,并于易分解样品等方面发挥作用。

## Abstract (en)

Microextraction is a rapidly developing sample preparation technology in the field of analytical chemistry, which is seeing widespread application. Accurate sample preparation can not only save time but also improve the efficiency of analysis, determination, and data quality. At present, sample pretreatment methods must be rapid, allow for miniaturization, automation, and convenient online connection with analytical instruments. To meet the requirements of green analytical methods and improve the extraction efficiency, microextraction techniques have been introduced as suitable replacements to conventional sample preparation and extraction methods. Microextraction using a packed sorbent (MEPS) is a new type of sample preparation technology. The MEPS equipment was prepared using microsyringe with a volume of 50-500 μL, including MEPS syringes and MEPS adsorption beds (barrel insert and needle, BIN), which is essentially similar to a miniaturized solid phase extraction device. The BIN contains the adsorbent and is built into the syringe needle. A typical MEPS extraction procedure involves repeatedly pumping the sample solution in two directions (up and down) through the adsorbent multiple times in the MEPS syringe. The specific operation course of MEPS includes conditioning, loading, washing, elution, and introduction into the analysis instrument. The conditioning process is adopted to infiltrate the dry sorbent and remove bubbles between the filler particles. The adsorption process is accomplished by pulling the liquid plunger of the syringe so that the sample flows through the adsorbent in both directions multiple times. The washing process involves rinsing the sorbent to remove unwanted components after the analyte is retained. The elution process involves the use of an eluent to ensure that the sample flows through the adsorbent in both directions multiple times, so that elution can be realized by the pumping-pushing action. The target analyte is eluted with the eluent, which can be directly used for chromatographic analysis. However, when processing complex biological matrix samples by MEPS, pretreatment steps such as dilution of the sample and removal of proteins are commonly required. At present, the operation modes of the MEPS equipment are classified into three types: manual, semi-automated, and fully automated. This increase in the degree of automation is highly conducive to processing extremely low or extremely high sample volumes. Critical factors affecting the MEPS performance have been investigated in this study. The conditions for MEPS optimization are the operating process parameters, including sample flow rate, sample volume, number of sample extraction cycles, type and volume of the adsorbent, and elution solvents. It is also necessary to consider the effect of the sample matrix on the performance of MEPS. The MEPS sorbent should be cleaned by a solvent to eliminate carryover and reuse. The sorbent is a core aspect of MEPS. Several types of commercial and non-commercial sorbents have been used in MEPS. Commercial sorbents include silica-based sorbents such as unmodified silica (SIL), C2, C8, and C18. Unmodified silicon-based silica is a normal phase adsorption material, which is highly polar and can be used to retain polar analytes. C18, C8, and C2 materials are suitable for reversed-phase adsorption, while SCX, SAX, APS, and M1 (C8+SCX) adsorbents are suitable for the mixed-mode and ion-exchange modes. Noncommercial sorbents include molecularly imprinted materials, restricted-access molecularly imprinted materials, graphitized carbon, conductive polymer materials, modified silicon materials, and covalent-organic framework materials. The performance of MEPS has recently been illustrated by online with LC-MS and GC-MS assays for the analysis of biological matrices, environmental samples, and food samples. Pretreatment in MEPS protocols includes dilution, protein precipitation, and centrifugation in biological fluid matrices. Because of the small sample size, fast operation, etc., MEPS is expected to be more widely used in the analysis of bio-matrix samples. MEPS devices could also play an important role in field pretreatment and analysis.

样品前处理技术是分析化学学科的重要研究内容。据统计,将一个原始的样品处理成可供仪器测定的样品状态,耗时约占整个分析时间的60%~70%^[1]^。正确的样品前处理不仅可以节约时间,还可提高分析测定效率和数据质量。目前样品前处理的趋势为快速、小型化、自动化、方便与分析仪器在线联用等。为适应针对样品前处理的相关要求,微萃取技术得以不断发展,并已广泛应用于各种基质的前处理中。微萃取技术主要可分为固相微萃取技术和液相微萃取技术^[2,3]^。微萃取技术详细的分类及发展见图1。

**图1:**
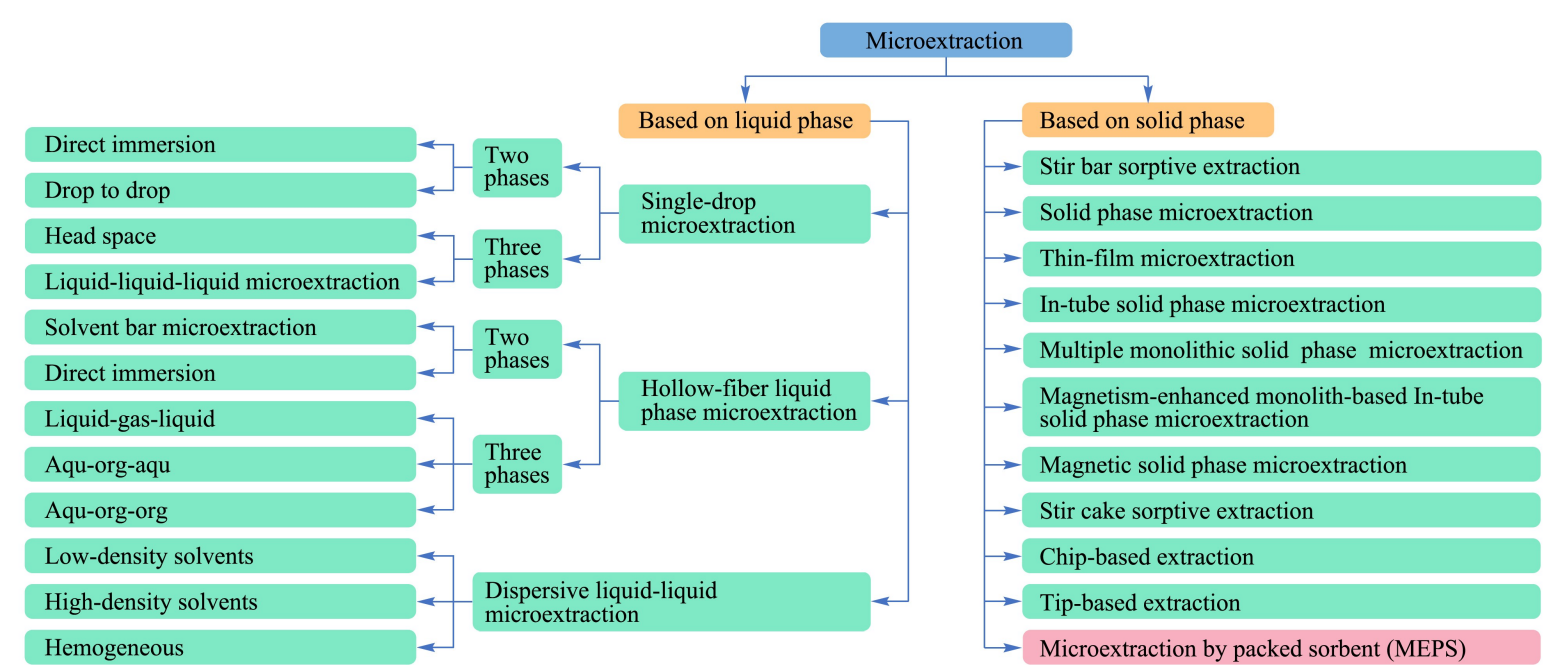
微萃取技术的分类总结

填充吸附剂微萃取(MEPS)技术是一种新型样品前处理技术,最早由瑞典的Abdel-Rehim于2004年提出^[4,5]^,该技术将微量吸附剂填充于微量注射器中制成萃取装置,实质是一种微型化的固相萃取装置,但在操作和使用方法上与传统固相萃取有所不同,其萃取过程基于微量的固体吸附剂,属于基于固体吸附剂的微萃取技术。2016年傅若农教授在综述中称MEPS为填充吸着剂微萃取^[6]^。样品经填充吸附剂微萃取后可直接使用气相色谱(GC)、液相色谱(LC)、质谱(MS)或离子迁移谱(IMS)等仪器进行后续分析。MEPS技术已经在生物、药物成分分析、食品安全及环境污染等多个领域得到应用。国内有关MEPS的综述尚未见报道。本文详细介绍了填充吸附剂微萃取技术所需的装置、吸附剂种类以及优化过程参数,并对其在药物及临床分析、食品以及环境分析中的应用进行了综述。

## 1 MEPS的设备和操作

典型的MEPS设备以体积50~500 μL的微量注射器为基础进行制备。MEPS设备与固相萃取柱之间的根本区别在于MEPS的吸附剂部分是直接集成到注射器中,而固相萃取柱却是单独的柱形萃取设备^[7]^。MEPS萃取设备包括两部分:MEPS注射器和MEPS吸附床(BIN),通常BIN中填充2~4 mg吸附剂^[8]^,萃取过程分为活化、上样、淋洗和洗脱4个步骤(见图2)。活化过程用来浸润干燥的吸附剂,去除填料颗粒之间的气泡。吸附过程通过拉动注射器液体推杆使样品多次双向流经吸附剂以完成吸附。淋洗过程是在分析物得到保留后,淋洗吸附剂去除不需要的组分。洗脱过程是用洗脱液通过抽-推的方式使样品多次双向流经吸附剂以实现洗脱^[9]^。

**图2:**
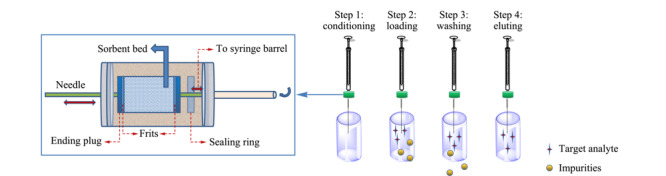
MEPS设备及操作方式

MEPS装置有手动、半自动和全自动3种形式^[10]^。手动模式可用微量注射器进行改装,改装过程为将微量注射器的推杆取出,放入一片多孔聚丙烯筛板,随后将2~4 mg固体吸附剂填入微量注射器推杆腔体,并填入另一片多孔聚丙烯筛板压紧。自动化设备主要利用截止阀和单向阀来实现自动上样、淋洗、洗脱、清洗等步骤(见图3a)。近年来出现了受控定向流动(CDF)^[7]^和μSPEed^[11]^等商用自动化设备。传统MEPS操作中,样品和溶剂通过同一通道上样和推出。对于与吸附剂相互作用较弱的目标分析物,可能会在样品萃取和洗涤步骤中部分洗脱并除去。CDF-MEPS设备如图3b所示,可使样品和溶剂通过独立的流动路径,从而更好地控制液体流动的方向,减少此步骤中目标分析物的损失。μSPEed是Eprep公司对MEPS做出新改进的市场化设备。如图3c所示,μSPEed设备^[12]^包含压力驱动单向止回阀,允许超低死体积连接和通过吸附剂床的单向流动路径,当柱塞向后拉时,不必通过吸附床而是绕开吸附剂,通过真空来实现抽吸,而推样品或溶剂时单向阀关闭,流经吸附剂实现洗脱。μSPEed设备允许样品及洗脱溶剂在恒定高压(最高11 MPa)条件下,单方向流经小粒径吸附剂,从而更有效地提取目标分析物。吸附剂部分高压接头设计为即插即用,可方便更换,这对于设备自动化而言尤为重要。虽然设备自动化后在处理样品量以及分析的平行性方面具有优势,但使用注射器自制MEPS,在吸附剂的应用开发方面仍然具有重要作用,杨柳课题组^[13]^曾报道过自制填充吸附剂微萃取设备萃取和检测水中的多氯联苯。

**图3:**
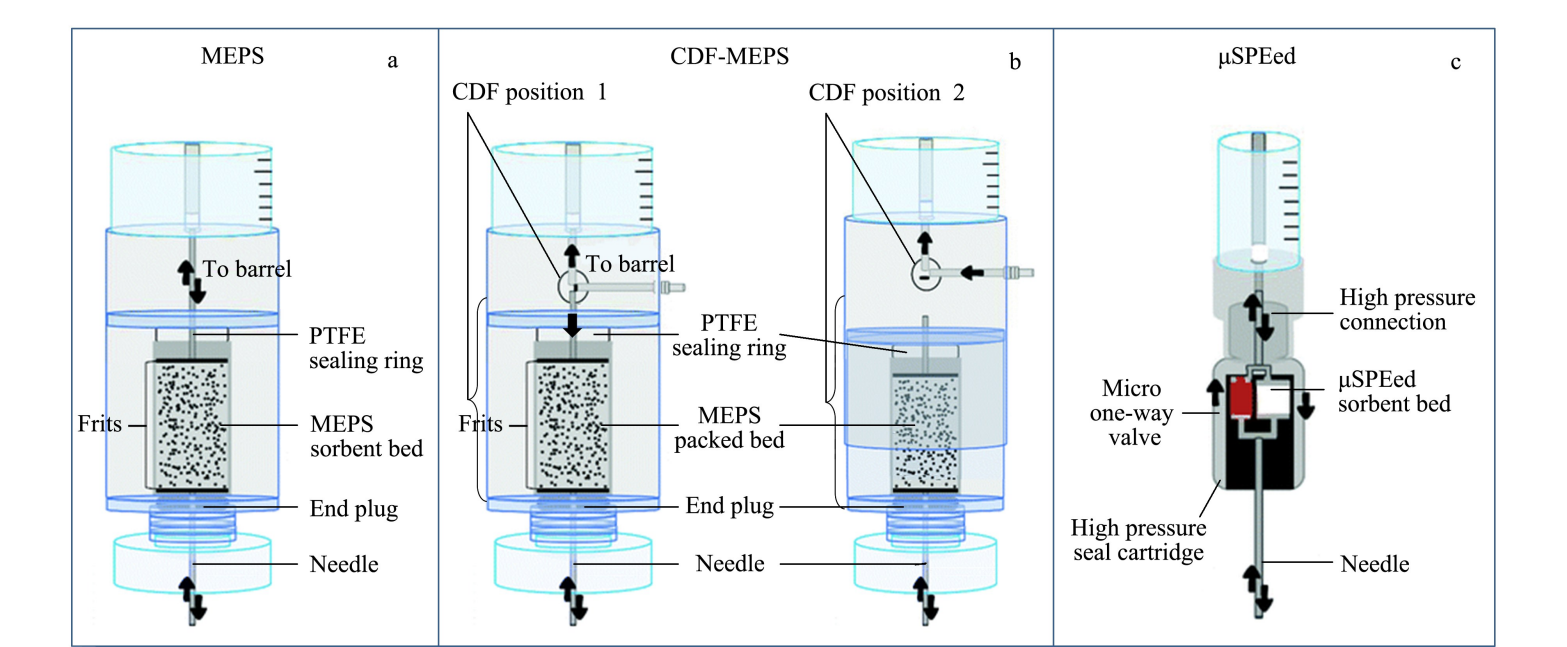
不同MEPS设备模式^[7]^

## 2 MEPS参数优化

为提高萃取效率,可对MEPS中的主要影响因素进行优化。影响MEPS萃取效果的因素主要有以下3个方面。

MEPS处理过程的影响。主要包括样品流速、样品量与样品萃取循环次数,吸附剂及淋洗、洗脱溶剂的种类和体积。例如在生物样品应用中,样品流速通常为10~20 μL/s,较低的样品流速有利于分析物与吸附剂之间更好地相互作用。萃取效率通常随着样品萃取循环次数的增加而增加,直到建立吸附平衡为止。虽然随着萃取循环次数的增加,提取效率会提高,但样品制备的时间也会增加。试验及应用过程中应当选择最小样品量和最少循环次数来获得对目标分析物最佳的萃取效果。通常血浆样品的萃取循环数为10~26次,对于尿液样品,为5~8次^[14,15,16,17,18,19]^。由于MEPS可同时萃取多种化合物,因此,必须建立一个折中方案,以达到最佳效果。优化过程可以采用单变量和多变量方法^[20]^。单变量为一次仅改变一个因素,其他因素保持不变。MEPS优化通常采用这种方法,但当因素数量增加时,实验量会剧增,此外少数情况下各因素之间可能会有相互作用。目前已经有响应面分析方法作为多变量法用于MEPS的优化过程^[21]^。吸附剂用量同样为萃取过程中一个重要的优化参数,根据材料对分析物的保留容量和特异性,吸附剂质量选择范围通常为2~4 mg。淋洗步骤中通常选择与活化过程相同的溶剂以去除杂质。洗脱溶剂对目标化合物应该有良好的溶解性,洗脱溶剂还需要考虑与检测技术相匹配,这样可以简化仪器进样前的样品处理。

样品基质对MEPS性能的影响。当处理血浆或尿液样本时,使用MEPS前必须经过1∶4体积的样品稀释。对于血液样品,通常的稀释倍数为20倍。通过MEPS消除基质效应对仪器的影响也很重要,如基质效应是电喷雾电离中一个众所周知的问题,如MEPS处理血液、血浆和尿液后使用ESI-MS检测时就需要考虑抑制基质中的离子。

样品的残留和重复使用的影响。MEPS可能重复使用的次数与样品的残留有关。通常经过清洗后MEPS的残留量少于0.1%。因此MEPS可重复使用几十次甚至几百次。

## 3 MEPS吸附剂

MEPS中最重要的部分是吸附剂^[22]^,其种类与固相萃取所用相似,吸附材料通过不同形式的作用机制以吸附保留分析物^[23]^。

### 3.1 商品化吸附剂

不同种类商品化的吸附剂已在MEPS设备中得到应用,如基于硅基的Silica、C18、C8、C2、SCX、SAX、APS、M1(C8+SCX)^[24]^,基于碳材料的Hypercarb和基于聚苯乙烯聚合物的SDVB、HDVB、retain-PEP、retain-CX、retain-AX吸附剂等^[25]^。

未修饰的硅基Silica为正相吸附材料,该材料为强极性,可用于保留极性分析物。C18、C8、C2材料均适用于反相吸附,保留机理主要基于分析物和萃取相之间的疏水相互作用等^[18]^。SCX、SAX、APS、M1(C8+SCX)吸附剂适用于混合模式和离子交换模式,SAX、SCX分别为强阴离子和强阳离子交换剂。M1(C8+SCX)具有双重保留机制,对生物液体中碱性化合物的选择性更高。基于碳材料的Hypercarb为多孔石墨碳材料,适用于对水溶性极性化合物的萃取,可用于从不同基质中提取农药。基于聚苯乙烯聚合物的SDVB、HDVB用于反相吸附,可保留非极性化合物。Retain PEP为尿素官能团改性的聚苯乙烯聚合物,用于反相和离子交换吸附,适用范围广泛,如用于提取生物流体中的药物和代谢物等。

### 3.2 非商品化吸附剂

非商用吸附材料是MEPS发展的重要部分,包括分子印迹材料及限进分子印迹材料、碳基材料、导电聚合物类材料、改性硅基材料及共价-有机骨架材料等。

分子印迹聚合物(MIPs)由于对目标分子具有特异性吸附效果,是开发新型吸附材料的重要方法,Moein课题组^[26]^报道了使用分子印迹填料作为吸附剂,经填充吸附剂微萃取测定肌氨酸,聚合物制备过程中使用了虚拟分子印迹聚合物方法,用具有与模板分子相似结构的化合物用作虚拟模板分子制备,减少了传统MIPs结构中模板泄漏的问题。限进材料主要用于吸附去除蛋白质等大分子,De Oliveira等^[27]^使用一种新型限进材料结合分子印迹聚合物作为吸附剂进行MEPS萃取,材料制备过程见图4。材料中增加了聚合物表面的亲水性,降低了生物液提取过程中蛋白质的保留,可用于测定人尿液样品中的雌激素。

**图4:**
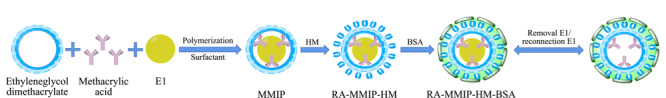
RA-MMIP-HM-BSA合成方案^[27]^

近年报道的碳基材料包括石墨碳材料(CarbonX COA)及CMK-3材料等。CarbonX COA吸附剂其石墨层面位于机械强度高的氧化铝基材外,且底物的存在和较弱的保留能力有助于保留分析物^[28]^。CMK-3是高度有序的碳骨架材料,具有纳米孔结构,其孔径分布窄,比表面积较大。Rahimi等^[29]^使用CMK-3碳材料作为吸附剂用于MEPS实验,研究中比较了CMK-3与普通活性炭的吸附效率,CMK-3的高孔隙率被认为是比活性炭更好的吸附剂。Khoshdel等^[30]^也在MEPS中将CMK-3材料作为吸附剂,根据实验结果可推测CMK-3中碳碳双键与生物酚的苯环之间的*π*-*π*相互作用可能会使萃取效果显著提高。基于碳材料的吸附剂可获得较好的重复使用性,但对于目标物的吸附选择性还需要进一步的深入研究。

导电聚合物纳米结构不仅具有高度*π*键共轭的聚合物链,还具有类金属的导电性等特点。聚苯胺纳米线具有高表面积、*π*-*π*相互作用等特征,具有较高的萃取能力^[31]^。聚吡咯/聚酰胺^[32]^也用于MEPS研究,该类导电聚合物具有高表面积、带*π*键共轭官能团和极性基团、表面光滑形态等特点。类似还有使用氧化石墨烯增强的聚酰胺材料用于MEPS等^[33]^报道。导电聚合物可通过静电纺丝的方法形成不同于球状的形态结构,将有助于提升吸附容量和吸附速率。

以硅球材料为基质,通过改性用于MEPS的研究也在发展,二氧化硅也可以通过不同的官能团功能化,如季胺或磺酸功能化后,用于离子交换萃取带电分子^[34]^。硅基材料的特点是在不同溶剂中具有高度稳定性,因此基于该材料进行修饰的方法是一种重要途径,如氨基丙基、氰基丙基修饰的硅材料。整体相硅材料相比传统的颗粒硅填充相,具有低背压、高传质速率和高渗透性的特点^[35]^。氨基丙基硅表面共价键合石墨烯作为吸附剂同样可避免石墨烯纳米片直接使用可能产生的高背压^[36]^。

官能化多孔共价有机骨架(COF)同样被用作吸附剂^[37]^, COF材料粒径较小,通常采用复合的方法,以适应MEPS的需求。MEPS中填充自组装的多孔共价有机骨架官能化聚(苯乙烯-二乙烯基苯-甲基丙烯酸缩水甘油酯)复合材料,用于提取水样中非甾体类抗炎药。这些非商品化的吸附剂在许多领域都被运用,但是考虑到各种使用条件及范围的限制,目前仍处于基础研究状态。

## 4 MEPS的应用

填充吸附剂微萃取技术结合多种分析仪器已经在药物分析、食品安全及环境监测等领域得到了应用。

### 4.1 生物样品

血液、血浆和尿液等生物样品为复杂基质,包含从小分子(如无机盐,磷脂)到大分子(如蛋白质)的各种化合物。当样品浓度过大或者黏度较大时,直接进行MEPS处理,会加重基质效应,还可能造成吸附剂堵塞。MEPS处理这些样品时,需要进行稀释样品(减小样品黏度)、调节pH值、去除蛋白质等预处理,然后根据检测目标物的性质选择仪器方法,以得到满意的检出限^[38]^。常规萃取方法,例如液液萃取(LLE)和固相萃取对不同生物样品基质中的大多数目标分析物也能得到良好的萃取效果。但传统萃取过程通常耗时长,并且需要大量有机溶剂和样品。Pautova等^[39]^报道了MEPS结合衍生化法提取血清中8种苯基羧酸的方法,用于确定危重病人血清样本中苯基羧酸的浓度。与液液萃取方法进行比较,MEPS方法无需重新进行悬浮和干燥处理。此外,MEPS可以洗涤基质,降低杂质进入色谱系统可能造成的损坏。样品制备时间仅需6 min,所需血清体积仅80 μL。Silveira等^[40]^使用MEPS结合LC-MS/MS,提取和测定内分泌干扰化学物质,与空气辅助液液微萃取(AALLME)、分散液相微萃取(DLLME)、液液萃取、固相萃取方法比较,MEPS所需时间更少(约6 min)。

部分生物样品使用填充吸附剂微萃取方法进行处理后,可在不经色谱分离情况下与质谱联用,这种方法可缩短临床应用中的分析时间。MEPS提取液直接注射到串联质谱系统中,可以快速测定生物材料中的药物和疾病标记物。Vl $\overset{ˇ}{c}$ ková等^[41]^应用MEPS技术萃取人尿液中的滥用药物安非他明和美沙酮,并结合质谱进行最终测定。尿液样品经过稀释,洗脱液直接从MEPS注射器注入串联质谱分析,此方法适合于临床和法医实验室大量样品的处理。MEPS与HPLC联用在生物化学分析中得到了应用,如分析测定人体基质中内源性皮质类固醇的分析方法^[42,43]^。Sorribes-Soriano等^[44]^使用MEPS-IMS方法用于测定口腔液中的甲酮。甲酮回收率为78%~91%,通过IMS获得的结果与通过HPLC-MS的结果之间没有差异。Martín Santos等^[45]^开发了程序升温汽化(PTV)结合MEPS-GC-MS方法用于提取测定唾液样品中的多环芳烃。检测过程中仅需将唾液样品放入小瓶,后续提取、分离和测定过程均可在线运行。多环芳烃的检出限仅为0.005~0.079 μg/L。表1中对比了MEPS方法与其他常规萃取方法的检测效果。

**表1:** MEPS与不同萃取方法在生物样品检测应用中的比较

Targets	Matrices	Extraction	Instrument	LOD/(ng/mL)		Ref.
Carnitine, acylcarnitines	urine	MEPS	UPLC-MS/MS	0.100		[46]
	urine	evaporation	CE	1.600		[47]
	humansera, rat tissue	SLE	UHP-HILIC- MS/MS	0.500	-5.000	[48]
Tetracycline residues	milk	MEPS	HPLC-MS/MS	0.030	-0.210	[49]
	milk	LLE	HPLC-MS/MS			[50]
	milk, eggs	FIL-NOSM	HPLC-UV	0.080	-1.120	[51]
	infant foods	SALLE	UPLC-MS/MS	0.050	-0.140	[52]
	beef	DLLME	HPLC-MS/MS	2.000	-3.600	[53]
	animal tissue	SPE	HPLC-MS/MS	0.500	-4.000	[54]
	honey	MF-SPME	HPLC-MS/MS	0.007	-0.017	[55]
Haloacetic acids	tap water, swimming pool water	MEPS	PTV-GC-MS	0.360	-1.200	[56]
		HF-LPME	GC-ECD	0.500	-3.000	
		SBME	GC-MS	0.020	-1.000	
		SDME	GC-MS	0.010	-0.200	
Endocrinedisrupting	urine	SPE	LC-MS/MS	0.100	-0.180	[57]
chemicals		LLE	LC-MS/MS	0.200	-2.000 (LOQ)	[58]
		DLLME	LC-MS/MS	0.005	-0.200	[59]
		AALLME	LC-MS/MS	0.010	-0.300	[60,61]
		SPE	UPLC-MS/MS	0.090	-0.370	[62]
		MEPS	UPLC-MS/MS	0.500	(LOQ)	[63]
		MEPS	LC-MS/MS	0.005	-0.100	[40]

SLE: supported liquid extraction; LLE: liquid-liquid microextraction; FIL-NOSM: functionalized ionic liquid-based non-organic solvent microextraction; SALLE: salting-out assisted liquid-liquidextraction; DLLME: dispersive liquid-liquid microextraction; MF-SPME: monolithicfiber-solid phase microextraction; HF-LPME: hollow-fibre membrane liquid-phase microextraction; SBME: solvent bar microextraction; SDME: single drop microextraction; AALLME: air-assisted liquid-liquid microextraction; UPLC: ultra performance liquid chromatography; UHP: ultra-high performance; HILIC: hydrophilic interaction liquid chromatography; PTV: temperature-programmed vaporizer.

除了较低的检出限外,所需样品体积为μL级也是MEPS的一大优势。Vl $\overset{ˇ}{c}$ ková等^[64]^对使用MEPS和固相萃取方法检测血浆中恩替卡韦的效果进行了对比。实验对两种不同的生物基质(血浆和血浆超滤液)进行了净化,与SPE洗脱液不同,MEPS洗脱液与随后用于恩替卡韦分析的亲水相互作用液相色谱(HILIC)流动相完全兼容,而SPE洗脱液必须在流动相中蒸发并重新配制。相比较于SPE需要500 μL样品量,MEPS方法的样品体积仅需要50 μL。由于血浆超滤液样品量有限,因此用量较少的MEPS技术适合于生物样品的分析,部分研究工作见表2。

**表2:** MEPS在生物分析中的应用

Matrix	Target	Sample volume/μL	Instrument	Ref.
Blood serum	phenyl carboxylic acid	50	GC-MS	[39]
Urine	amphetamine	100	GC-MS	[65]
Saliva	dichloropane	100	IMS, GC-MS	[66]
Aqueous humor	dexamethasone disodium phosphate and dexamethasone	50	LC-MS/MS	[67]
Urine	*trans*,*trans*-muconic acid	100	MIMEPS-HPLC-UV	[68]
Oral fluid	new psychoactive substances	100	UPLC-MS/MS	[69]
Plasma	dietary phenolic acid	50	GC-MS	[70]
Blood serum	antisense oligonucleotide	50	UPLC	[71]
Urine	mandelic acid	100	MIMEPS-HPLC-UV	[72]
Urine	cocaine and its metabolites	5	MEPS-DART-TOF	[73]
Urine	metabolite	70	GC-MS	[74]

IMS: ion mobility spectroscopy; MIMEPS: the combination of a molecularly imprinted polymer and microextraction by packed sorbent; UV: ultraviolet; DART: direct analysis in realtime; TOF: time of flight.

### 4.2 食品样品

固相萃取方法在食品成分测定与安全分析中十分常见,但固相萃取方法吸附过程中样品流速通常较慢,如SPE处理5 mL样品时,以1 mL/min的流速处理,完成洗脱大概需要9 min,而MEPS却可以在0.75 min内完成。与传统SPE方法相比,MEPS可以实现在极短时间内拥有相对较高的浓缩系数。

MEPS已用于食品相关的前处理中。Perestrelo等^[75]^使用MEPS与超高压液相色谱-光电二极管阵列检测器(UPLC-PDA)结合,测定马德拉酒中的呋喃衍生物。将MEPS-UPLC-PDA测定的结果与顶空固相微萃取-气相色谱-质谱法(HS-SPME-GC-MS)相比较,在灵敏度和重现性等方面显示出更好的效果。Rahimi等^[76]^使用MEPS萃取结合HPLC测定果汁样品中鞣花酸。通过MEPS方法,在纳米多孔二氧化硅吸附剂上富集分析物。与SPE方法相比,MEPS方法减少了吸附剂量(2 mg)、有机溶剂用量(0.3 mL)和样品量(250 μL)。自动化MEPS设备在简化提取过程的同时减少了手动方法带来的误差。该方法比以前报道的提取鞣花酸的方法更快、更简单和更经济。MEPS技术不仅可有效从液体(如苹果酒或葡萄酒)中提取分析物,同样也可用于固态样品的提取。如Paris等^[77]^报道使用MEPS萃取苹果中的多环芳烃。该方法用乙醇进行超声辅助溶剂萃取(UAE),然后使用吸附剂填充微萃取进行富集。UAE-MEPS方法样品前处理时间仅为HS-SPME方法和UAE-SPE方法的1/2,且与UAE-SPE方法相比,UAE-MEPS方法样品制备所需的有机溶剂量少,更为绿色环保。

MEPS同样应用于食品成分中污染物和农药的采集。例如李新培等^[78]^合成了一种金纳米颗粒(AuNPs)复合材料,用作MEPS吸附剂,萃取并检测玉米样品中4种三嗪类除草剂。Abolghasemi等^[79]^采用纳米结构星形聚噻吩用作MEPS吸附剂,萃取牛奶和果汁样品中氯芬太嗪农药。此外还有实验表明,MEPS不仅可用于富集单一种类目标分析物,亦可利用MEPS进行多种类成分残留的提取。Di Ottavio等^[80]^利用MEPS-UPLC-MS/MS方法萃取检测小麦粉中25种农药和杀真菌剂残留。Montesano等^[81]^利用MEPS-LC-MS/MS同时测定几种不同类别非法药物口服液。事实证明,这种方法仅需少量样品(120 μL),且MEPS吸附剂可重复使用约100次而性能不损失。此外,MEPS还可与传感器或生物传感器联用,用于测定具有挑战性样品基质(例如小麦粉)中的分析物。Capoferri等^[82]^通过循环伏安法(CV)在玻璃碳电极(GCE)的表面上实现了乐果-聚吡咯MIP膜,然后利用MEPS结合使用MIP传感器检测乐果。该方法使用成本低,易于使用,可以为开发现场传感测试分析方法开辟新思路。

### 4.3 环境污染物

MEPS已用于提取环境水样品中的药物、农药、多环芳烃和其他有机污染物。使用农药是保护农作物免受病虫害必不可少的措施,但农药的过量使用也会造成环境污染^[83]^。因此从环境水样中提取农药并对其进行监测十分重要。Taghani等^[84]^以天然纳米硅藻作为吸附剂,采用MEPS-GC-MS方法对水样中3种有机氯农药进行萃取测定。该方法具有较低的检出限(0.02~0.13 μg/L)。Saraji等^[83]^以聚硅氧烷网络和纳米黏土颗粒的复合物为吸附剂,使用MEPS-电晕放电-离子迁移谱仪(CD-IMS)检测河流、油井和农业用水中的二嗪农,这是首次报道将MEPS-CD-IMS用于分析检测。经过40次重复实验,吸附剂聚硅氧烷网络/纳米黏土颗粒复合物的吸附效率仅下降了8.3%。Mousavi等^[85]^以合成的含咪唑骨架的介孔有机硅为MEPS吸附剂,用于萃取苯氧酸除草剂、多环芳烃和氯酚,该吸附剂可重复使用大约80次。农药存在于环境中,进入人体后会对人体造成巨大伤害。Santos等^[86]^以C18为吸附剂,采用MEPS-GC-MS/MS方法同时测定血样中6种有机磷农药。该方法具有良好的定量限(2.5 μg/mL(乙基-谷硫磷))且使用样品量小(150 μL)。Klimowska等^[87]^采用MEPS-大体积进样(LVI)-GC-MS测定人尿中5种拟除虫菊酯代谢物。所需样品量仅为400 μL,且检出限为(0.06~0.08 ng/mL)。作为一种高通量的快速萃取方式,MEPS同时用于受污染水体中药物的采样与富集,例如布洛芬、酮基布洛芬^[88]^、芳香胺^[89]^、大环麝香化合物^[90]^、卤代乙酸^[56]^、多环芳烃^[91]^、磺胺类药物^[92]^、喹诺酮类药物^[93]^等。Caballero-Díaz等^[94]^研究了MEPS萃取江水样品中的麝香酮,使用表面增强拉曼(SERS)进行检测,由于所使用的拉曼光谱仪与MEPS一样具有便携性,因此该方法适用于现场监测。Ferreira等^[95]^则使用MEPS在不需色谱分离的条件下直接结合MS,在线完全自动化的前提下对3-甲基-1-丁醇等目标分析物进行提取,缩短了分析时间。该方法同传统的GC-MS联用方法^[96,97]^进行比较,重复性和再现性均获得良好的结果。MEPS还用于采集河流水体中的硝基爆炸物。Grueiro Noche等^[98]^以C18为吸附剂,将MEPS与GC-MS联用,开发了一种简单、快速的用于硝基炸药的分析方法。与常规LLE、SPE等萃取方法相比,该方法减少了样品的制备和分析时间,且能获得较低的LOD值(0.014~0.828 ng/mL)。

## 5 展望

本文详细介绍了MEPS的设备构成、萃取操作流程和各种类型的吸附剂及其应用,分析了影响MEPS萃取效率的主要影响因素。就近几年MEPS在生物、食品、环境水样分析检测中的研究与应用进展做了简要介绍。填充吸附剂微萃取技术作为微型化萃取技术,适用范围广,可利用其样品体积小、操作快速等特点,同时文中潜析了其在现场使用、生物样品基质分析、新材料研发等方面不断发展的趋势。MEPS未来的研究和应用主要包括新吸附材料的开发、结合多样的分析仪器实现小型化现场检测等。从MEPS可联用的仪器来看,已经从传统的气相色谱、液相色谱、质谱等仪器,不断扩展到离子迁移谱、拉曼光谱等现场便携式仪器,使现场快速前处理及检测的手段更加丰富。例如在环境样品中,该技术可与现场便携仪器联用,未来将有望在现场进行快速检测,并于易分解、降解样品的检测等方面发挥作用。MEPS同样存在一些局限性,例如黏性或高浓度样品未事先稀释时,吸附剂部分很容易堵塞,吸附剂粒径过细也容易造成堵塞。MEPS的发展在很大程度上取决于吸附剂的发展,因此随着新型吸附剂不断开发,MEPS的应用也将更加广泛。
